# Highly heterogenous humoral immune response in Lyme disease patients revealed by broad machine learning-assisted antibody binding profiling with random peptide arrays

**DOI:** 10.3389/fimmu.2024.1335446

**Published:** 2024-01-22

**Authors:** L. Kelbauskas, J. B. Legutki, N. W. Woodbury

**Affiliations:** ^1^ Biodesign Institute, Arizona State University, Tempe, AZ, United States; ^2^ Biomorph Technologies, Chandler, AZ, United States

**Keywords:** peptide array, antibody profiling, machine learning, predictive modeling, Lyme disease (LD), humoral immune response

## Abstract

**Introduction:**

Lyme disease (LD), a rapidly growing public health problem in the US, represents a formidable challenge due to the lack of detailed understanding about how the human immune system responds to its pathogen, the *Borrelia burgdorferi* bacterium. Despite significant advances in gaining deeper insight into mechanisms the pathogen uses to evade immune response, substantial gaps remain. As a result, molecular tools for the disease diagnosis are lacking with the currently available tests showing poor performance. High interpersonal variability in immune response combined with the ability of the pathogen to use a number of immune evasive tactics have been implicated as underlying factors for the limited test performance.

**Methods:**

This study was designed to perform a broad profiling of the entire repertoire of circulating antibodies in human sera at the single-individual level using planar arrays of short linear peptides with random sequences. The peptides sample sparsely, but uniformly the entire combinatorial sequence space of the same length peptides for profiling the humoral immune response to a *B.burg.* infection and compare them with other diseases with etiology similar to LD and healthy controls.

**Results:**

The study revealed substantial variability in antibody binding profiles between individual LD patients even to the same antigen (VlsE protein) and strong similarity between individuals diagnosed with Lyme disease and healthy controls from the areas endemic to LD suggesting a high prevalence of seropositivity in endemic healthy control.

**Discussion:**

This work demonstrates the utility of the approach as a valuable analytical tool for agnostic profiling of humoral immune response to a pathogen.

## Introduction

Lyme disease (LD) is the most prevalent tick-borne disease in the United States with an estimated 476,000 new cases annually (1, 2). The pathogen causing the majority of LD cases in the US, *Borrelia burgdorferi (B.burg.)*, a spirochete bacteria, is spread by a bite from an infected *Ixodes scapularis* tick. While correctly diagnosed LD can be treated with antibiotics, the disease diagnosis represents a formidable challenge due to the lack of reliable diagnostic tools. Clinical diagnosis of LD is limited as it relies upon the patient presenting with a characteristic “bullseye” rash (*Erythema migrans*, EM). However, a large portion of LD patients either do not present with the rash at all or exhibit differing appearance of the rash complicating the correct diagnosis that is largely dependent on skill and experience of the diagnosing physician. The only molecular diagnostic tool for LD currently recommended by the American Centers for Disease Control and Prevention (CDC) is the Standard Two-Tier Test (STTT) (3) and, more recently, Modified Two-Tier Test (MTTT) (4), a serological test that constitutes the detection of LD-specific adaptive humoral immune response in the form of a panel of LD-specific antibodies in the patient’s serum. However, the test suffers from low sensitivity and a high false-positive rates, especially in the early stages of the disease (5). Person-to-person variability in both immune response timing and antigenic targets as well as intricate interplay between *B.burg.* and host immune system have been suggested as main factors for the poor test performance. Following tick bite, *B. burg.* is well equipped to evade the complement and adaptive immune response and avoid triggering the generation of specific antibodies. The bacterium benefits initially from immunosuppressive *Ixodes* saliva proteins allowing it time to upregulate genes needed to survive the mammalian host environment. Expression of surface proteins which bind complement system regulatory proteins results in the short-term inhibition of opsonization and subsequent damage to the bacterium (6, 7). Interference with the complement system further shields *B. burg.* from the adaptive immune system by removing triggers such as C3b that recruit immune cells to the infection site (8). Within a single infection, there is a progression of surface protein expression starting with OspA at inoculation, transitioning to OspC as infection is established and finally the antigenically shifting VlsE as disease progresses (9–12). The challenges presented by the *B. burg.* survival strategies which shift with disease progression are also reflected in the lack of accurate serologic diagnostic tools for LD. This suggests that the complexity of the *B. burg.* behavior in human hosts combined with the person-to-person variability in immune response requires new approaches to explore the host immune response to *B. burg*. in its broadest sense and in an agnostic way. As a result, to develop more robust serologic diagnostics and to capture the individual immune responses in patients, it appears necessary to profile humoral immune response at the single-patient level and without an *a priori* bias towards specific antigens.

Broadly profiling of the humoral immune system arm represents unique challenges. It is estimated that at baseline the number of unique antibodies (Ab) in a person’s repertoire is on the order of 10^12-15^ making studies to experimentally interrogate the binding profile in its entirety prohibitively expensive and impractical. Studies of Ab response are further complicated by substantial inter-individual variation both in terms of immune system homeostasis and response even to the same challenge (e.g. vaccination or infection with a pathogen) reported in emerging research (13–16). While current technological advances have made broad Ab profiling possible, significant limitations remain. For example, bacterial phage display (17–19), a widely adopted technique for determining epitopes and mimotopes of Ab binding, is a high-throughput approach based on the expression of large (~10^7^-10^9^) libraries of random peptides on the surface of bacterial phages followed by a series of stringent washing and amplification steps to select for phages expressing Ab-binding peptides. Phage immunoprecipitation sequencing (PhIP-Seq) that is based on combining phage display of a synthetic representation of complete proteomes with immunoprecipitation and high-throughput sequencing (20) has been demonstrated to enable autoantigen discovery in the human proteome and antigen detection in the virome (21). Antibody binding epitope mapping (AbMap) is another technique that combines phage display with next-generation sequencing (22). Due to the stringent selection, however, the methods are strongly biased towards peptides that bind antibodies with high affinity and can miss other, clinically relevant but lower affinity antibodies.

The advent of peptide microarray (PM) technology has enabled inquiries into a broad variety of questions in biomolecular recognition, primarily focusing on protein-protein and peptide-protein interactions (23–27). Ab reactivity profiling (epitope mapping) has been one of the main research fields where the use of this technology resulted in a number of impactful discoveries (23, 27, 28) demonstrating the utility of the approach in discovering biologically relevant information. When used in combination with random peptide libraries, the approach offers a way for interrogating the combinatorial binding space agnostically without any *a priori* knowledge about the pathogen underlying a particular immune response. Work conducted by this lab has demonstrated that it is possible to characterize the binding profiles of different proteins by combining binding information collected with an array of ~125,000 random peptides that sparsely and evenly sample an entire combinatorial space of ~10^12^ peptides with machine learning (ML) methods (29). This finding implies that, at least in theory, one could explore the binding profile of a polyclonal response by sparsely and evenly sampling the entire binding space with randomly generated peptide libraries and obtain relevant information about the circulating Ab repertoire. This hypothesis is supported by previous studies directed at Ab binding profiling using solid-phase planar PMs in sera of patients diagnosed with a number of different infectious diseases to robustly distinguish circulating Ab repertoires elicited by the humoral immune response to various pathogens (30–43). In contrast to this study, these efforts used a purely statistical basis to distinguish Ab binding profiles, without taking into account the sequence information contained in the library peptides the antibodies bound to. A more recent study performed in this lab focused on 5 human pathogens, 4 viruses and a trypanosome (Hepatitis B and C, Dengue Fever, West Nile Virus and Chagas disease) and explored the possibility of relating the peptide sequence information to the binding of total IgG from serum on the arrays. By utilizing a neural network (NN) model to relate peptide sequence to its binding strength, a marked improvement not only in classification performance but also in robustness against noise was demonstrated (44). The study corroborated the notion that despite very sparse sampling of the entire combinatorial peptide sequence space (~10^5^ peptides sampling a space of ~10^12^ total possible peptides), there is enough information to successfully model polyclonal Ab profiles in response to the 5 different pathogens. Furthermore, the ability of the approach to predict the cognate epitope of a monoclonal antibody using models trained on binding to near random sequences was reported (45). Very sparse, near random sampling of the entire combinatorial space allows one to generate a statistically accurate model for the binding to any other random samples of sequences. These findings suggest that despite the limitation one is facing with linear peptide arrays to likely miss structural epitopes, Ab binding to linear peptides contain enough information about the disease-specific humoral immune response to reliably distinguish different diseases and recognize short sequences they interact with in proteins.

This study is focused on interrogating and modeling the human humoral immune response to the *B. burg.* bacterium. As an alternative to a full-scale profiling of the circulating Ab repertoire, this work profiles and compares the binding of the circulating Ab repertoire in LD patients, healthy controls from both LD endemic and non-endemic geographic areas in the US, and patients diagnosed with diseases that have similar etiology to LD by sparsely sampling the entire combinatorial binding space. The sampling is performed with a set of peptide sequences that broadly cover the entire binding space, but whose number is orders of magnitude smaller than the number of all possible sequences. Using ML methods to model the peptide sequence-binding relationship of binding to the peptides on the array, it is then possible to map the information learned from the array-based serum Ab binding onto potential antigens from the *B. burg.* proteome. The working hypothesis is that binding of Abs raised in response to *B. burg.* infection to their respective target antigens can be modeled by measuring generally weaker binding to peptides with properties similar to the targets (mimotopes). If true, one should be able to reconstruct reasonably well the overall binding profile of a patient’s Ab repertoire by sparsely sampling, in a nearly random fashion, the entire combinatorial binding space. In contrast to the previous studies performed by this group, this work extends the utility of the approach through associating binding information measured on the arrays with biologically relevant insight by predicting Ab reactivities to the entire *B. burg.* proteome. The antigen identification method used in this study builds upon Ab profiling but integrates a sequence-binding relationship developed via ML models based on the NN approach to relate binding data with the peptide sequence. Due to the near-random nature of the peptide sequences, the peptide array can be thought of as a reduced, sparsely sampled representation of the entire binding space of a patient’s Ab repertoire binding to, on average, 9-10-mer peptide sequences with a total of 20^10^ possible peptides, and one can use ML/NN approaches to “learn” a quantitative relationship between the sequence of amino acids and Ab binding (29). The NN approach effectively provides a way to integrate the measured binding data into a single model that reliably captures the observed peptide sequence-binding relationship. The resulting computational NN models allow one to predict binding of any possible peptide sequence not represented in the library. Importantly, these computational models can also be built for each individual donor and, when applied to tiled protein sequences from the entire *B. burg.* proteome or any other pathogen proteome, including serotype specific sequences, provide a complete immune response map for each patient. This enables the identification of epitopes predicted to have differential binding between LD patients, healthy controls and disease with similar clinical symptomology on an individual patient basis.

Of course, peptide arrays are inherently biased towards Ab binding to linear, contiguous epitopes, and provide only partial binding to structural epitopes. However, despite this limitation, the approach offers unprecedented levels of detail into overall reactivities of a patient’s circulating antibodies with the potential to provide new insight into immune response and discover new antigenic targets associated with LD. Furthermore, the method enables relatively simple and rapid profiling of hundreds to thousands of patient samples reliably and reproducibly at a relatively low cost.

## Results

The binding assays were performed as described previously (44, 46). Briefly, human serum was diluted 1:625 in mannitol and incubated on peptide arrays at 37^°^ C. The dilution factor was determined over a number of earlier studies performed by this group. It was established that this dilution provides the best coverage of the detector dynamic range in terms of binding signal distribution. Following incubation and washing steps, the arrays were incubated with a fluorescently labeled goat anti-human IgG secondary Ab. The arrays were then washed, dried under nitrogen and imaged on a fluorescence microscope. Each image was analyzed and fluorescence intensities for each peptide sequence were extracted for further analysis. Data quality was assessed utilizing a set of replicate control peptides with identical sequences distributed in a random fashion across the entire array. Samples that showed a coefficient of variation in intensity of these probes of >0.2 were excluded from further analysis. This resulted in the exclusion of an average of 5-10% of samples per cohort (**Table 1**).

**Table 1:** Donor cohort breakdown.

Category	Disease	Number of samples
Cases	Lyme (seropositive)	99
	Lyme (seronegative)	91
Controls	Endemic healthy	110
	Non-endemic healthy	64
Look-alike diseases	Alcoholic liver disease	10
	Anti-nuclear antibodies	14
	Babesia	14
	Chlamydia	12
	Epstein-Barr virus	11
	Influenza	12
	Rheumatoid arthritis	14
	Syphilis	19

The binding data was analyzed in two different ways. The first method was based on the measured Ab binding to the peptide arrays and was used to assess how well can one differentiate between the different diseases (LD *vs*. look-alike diseases), disease states (seropositive *vs*. seronegative LD) and the healthy controls. To this end, performance of classifiers trained on the array data to distinguish the different sample types was assessed and compared. The main question this type of analysis addresses is whether the sparse sampling of the binding space embodied by the peptide arrays captures enough disease-specific information about LD to enable its robust differentiation from other diseases and healthy controls. This analysis does not take into account the peptide sequence-binding relationship and relies instead on purely statistical approaches to select and combine a set of array peptides with the most differentiating power. The second method is built around predictive NN models trained on the array binding data to project a learned sequence-binding relationship onto the *B. burg.* proteome. The main objective of this analysis was to associate the information captured on the peptide arrays with biologically relevant, disease-specific insights and identify potential immunogenic targets the patient’s antibodies are reactive against.

### Similar Ab reactivity distributions between LD and healthy controls

**Figure 1:**
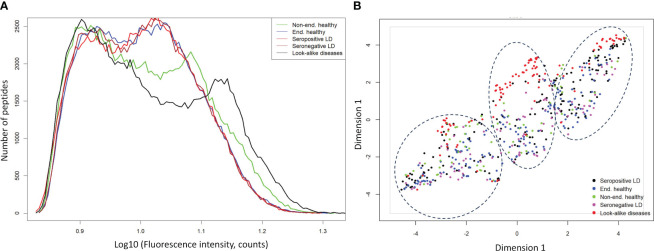
Ab binding profiles measured on the diverse peptide library of 5 different donor cohorts representing healthy individuals from non-endemic LD areas, healthy donors residing in LD endemic areas, seropositive LD patients, seronegative, clinically diagnosed LD patients, and look-alike diseases with etiology similar to LD. **(A)** Comparison of binding intensity distributions among the different donor cohorts reveals relatively minor differences among the endemic healthy donors and LD patients but shows strongly differentiating profile between the look-alike diseases and the other 4 cohorts. The fluorescence intensities of each peptide on the array were averaged over the entire corresponding cohort. **(B)** UMAP representation of the measured fluorescence intensities with each data point representing an individual donor. The plot shows little separation among LD and endemic healthy cohorts but suggests marked differentiation between the look-alike diseases and LD and healthy cohorts.

To evaluate whether gross differences in the Ab binding profiles between the donor cohorts as measured on the peptide arrays exist, peptide binding intensity profiles were compared between the different types of donors (**Table 1**). The peptide binding intensities were log10 transformed to make the binding intensity distributions more normal-like. For comparison purposes, the binding intensity values for each peptide and each donor sample were normalized by calculating the ratio of the peptide intensity and the median intensity value of the peptide for the entire corresponding cohort. For all 5 cohorts the binding intensity distributions show 2 distinct peaks, one centered at the low end of the binding range of 0.50-0.95 and the second peak representing stronger binding peptides in the 1.05-1.20 range (**Figure 1A**). The weakly binding peptides depicted by the first peak for all 5 cohorts show a substantial overlap and essentially identical binding intensity distributions. In comparison, the peptides in the second peak that correspond to stronger Ab binding show greater differences between the cohorts both in terms of peak location and height among the cohorts. The look-alike diseases show the most differentiating second peak in the binding intensity profile compared with the other 4 cohorts. Both the location and the height of this peak are markedly different, with the peak located in ~1.1-1.2 intensity range and up to 50% lower peak height. Similarly, the non-endemic healthy cohort exhibits largely different distribution of the stronger binding peptides from the other donor types. Interestingly, both LD cohorts (seropositive and seronegative) and the endemic healthy donors are almost indistinguishable from one another. Overall, compared with the other cohorts, the look-alike binding intensity distribution suggests a substantially stronger, broader Ab reactivity with the distinct second peak well differentiated from the first peak. In contrast, while the two LD cohorts and the endemic healthy donor group are very similar, all three are distinctly different from the look-alike and healthy non-endemic donors. The relative invariance of the first peak in the distributions of all 5 donor cohorts suggests that there is little Ab reactivity against a substantial number (~50-60%) of peptides on the array. The differences between the cohorts are contained mostly in the stronger binding peptides represented by the second peak in the distribution that contain most of the disease-specific Ab binding information.

To further assess the ability to differentiate the five different types of donors based on Ab binding to array peptides, the peptide binding data was transformed using the Uniform Manifold Approximation and Projection (UMAP) method (47). The method allows mapping of high dimensional data to a low dimensional space, while retaining most of the cohort-specific information. The peptide binding data projected onto a 2-dimensional space using UMAP is shown in **Figure 1B**. The plot reflects the general trends observed in the intensity distributions (**Figure 1A**). The look-alike donors are most differentiated from the rest of the donor types, followed by the non-endemic healthy controls. In contrast, the two LD cohorts and the endemic healthy controls are largely indistinguishable from one another, with the individual donors interspersed among the three cohorts. This finding corroborates the main result observed in **Figure 1A** with grossly similar binding profiles among these cohorts. However, the UMAP representation revealed another important point. The data point distribution indicates the presence of at least 3 potential clusters (dashed ellipses in **Figure 1B**) suggesting underlying sub-populations of the donors within the cohorts. Furthermore, the fact that the clusters are not cohort-specific implies that there is strong apparent donor-to-donor variability in Ab binding to the peptides even within the same cohort. In summary, this cohort-level analysis indicates that while the non-endemic healthy controls and look-alike diseases show marked differences in Ab binding profile between one another and the LD and non-endemic healthy controls, both LD cohorts and the endemic healthy controls exhibit highly similar Ab binding to the array peptides profile.

### Sample classification between cohorts using measured binding intensity to the array peptides

In addition to comparing the binding profiles at the cohort-level, disease classification of the different cohorts was explored using the measured Ab binding to the array peptides. In previous studies, this has been done directly using the binding values from the array peptides (35, 36, 42–44). In this case (a so-called “immunosignature”), feature selection is performed, often using t-tests to select which peptides best distinguish cohorts as a first step in developing classifier models.

**Figure 2:**
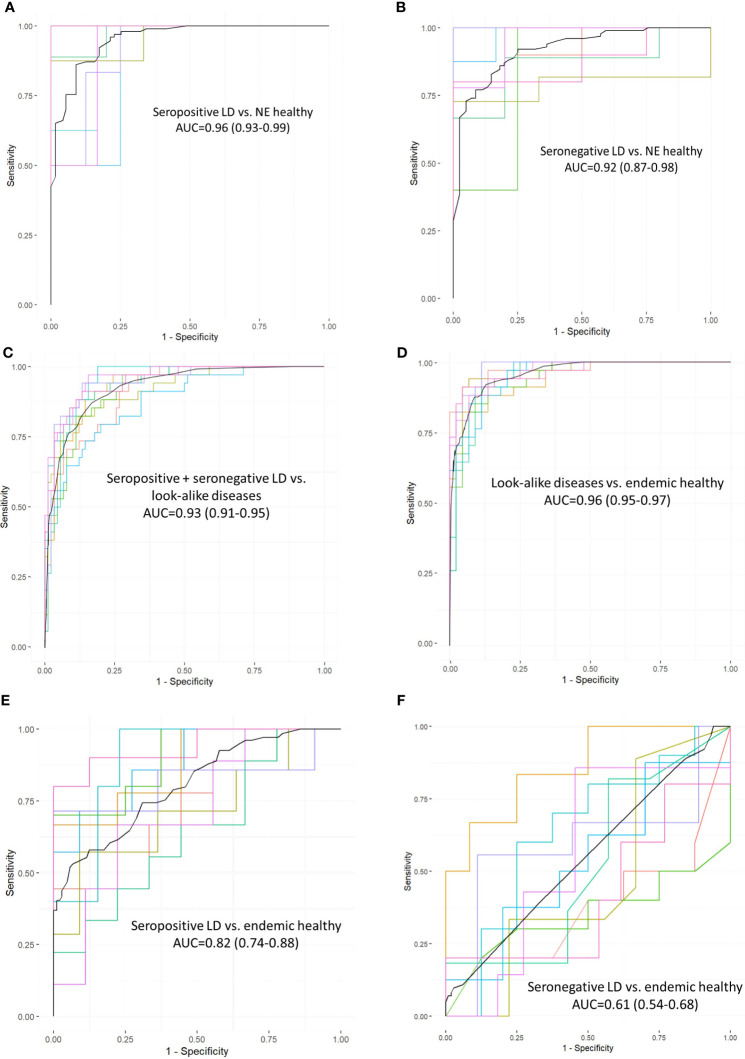
Receiver operating curves (ROC) representing performance of classifiers trained on measured Ab reactivity against array peptides to differentiate between the different donor cohorts. The classifiers were developed using the XGBoost approach with a 10-fold cross-validation utilizing randomly selected 10% of the samples for validation. Area under the curve (AUC) values are given along with the 95% confidence intervals indicated in the brackets. ROC curves represent classification performance between the following cohorts: **(A)** seropositive *vs*. healthy donors from non-endemic (NE) areas. **(B)** clinically diagnosed, seronegative LD *vs* healthy donors from NE areas. **(C)** combined seronegative and seropositive LD *vs*. diseases with look-alike symptoms, **(D)** look-alike diseases *vs*. endemic healthy controls, **(E)** seropositive LD *vs*. endemic controls. **(F)** seronegative LD *vs*. endemic controls.

To assess whether the different cohorts could be distinguished using the measured binding profiles from the peptide array, models were trained for pair-wise classification of the different donor types. The main question addressed here is how well binding to a library of peptides with near-random sequences can recapitulate clinical and serological differences between the different types of donors. To accomplish this, a number of classifiers were trained to distinguish between LD, look-alike diseases and normal controls. The Extreme Gradient Boosting (XGBoost) method was combined with the Random Forest (RF) approach for classifier model development. Due to the inherent ability of RF to perform feature selection during training, no features were selected *a priori*. The training was performed 10 times using 10-fold cross validation with randomly selected 10% of the samples as a hold-out dataset. The classification results presented as Receiver Operating Curves (ROC) and their Area Under the Curve (AUC) are shown in **Figure 2**. The results indicate robust differentiation between the seropositive LD cohort and the non-endemic healthy controls (**Figure 2A**) with an AUC of 0.96 (95% CI: 0.93-0.99). A slightly lower classification performance is observed between the clinically diagnosed, seronegative LD and the non-endemic controls (**Figure 2B**) with AUC=0.92 (95% CI: 0.85-0.99). Both ROCs show high sensitivity values at high specificity (0.75 sensitivity at 0.90 specificity). However, a more detailed comparison of the ROC characteristics shows that the classification performance of the seronegative LD cohort shows markedly lower specificity at sensitivity of 1. Here, while the seropositive LD *vs*. healthy controls classification reaches 0.5 specificity at a sensitivity of 1, in case of seronegative LD *vs*. healthy controls specificity drops to 0.25. This suggests a higher similarity of the circulating Ab binding profiles between the seronegative (clinically diagnosed) LD and the non-endemic healthy donors as compared with the seropositive LD *vs*. non-endemic controls. In comparison, the performance of a classifier trained to distinguish between the look-alike diseases and a combined cohort of seropositive and seronegative LD patients (**Figure 2C**) shows robust classification with an AUC=0.93 (95% CI: 0.91-0.95). This finding is especially relevant from the perspective of differential diagnosis, i.e. distinguishing between LD and a number of diseases with identical or similar clinical manifestations. This comparison is important from the perspective of validation the ability of the approach to distinguish between clinically different types of individuals. Similarly, look-alike diseases show strong differentiation from the endemic healthy controls (**Figure 2D**) with an AUC of 0.96 (95% CI: 0.95-0.97). Interestingly, a substantial drop in classification performance was observed when comparing either the seropositive LD or seronegative LD *vs* endemic healthy controls with a resulting AUC of 0.82 (95% CI: 0.74-0.88) (**Figure 2E**) and 0.61 (95%CI: 0.54-0.68) (**Figure 2F**), correspondingly. In parallel to the findings with the binding profile distributions, this result suggests a markedly more similar Ab reactivity profiles between the two LD cohorts and the endemic healthy controls as compared with the look-alike diseases and non-endemic healthy controls. These results also demonstrate that binding profiles measured with a library of peptides of diverse sequences contain relevant information enabling robust differentiation between the LD donors and look-alike diseases and non-endemic healthy controls.

### Binding motif discovery with peptide array data

**Figure 3:**
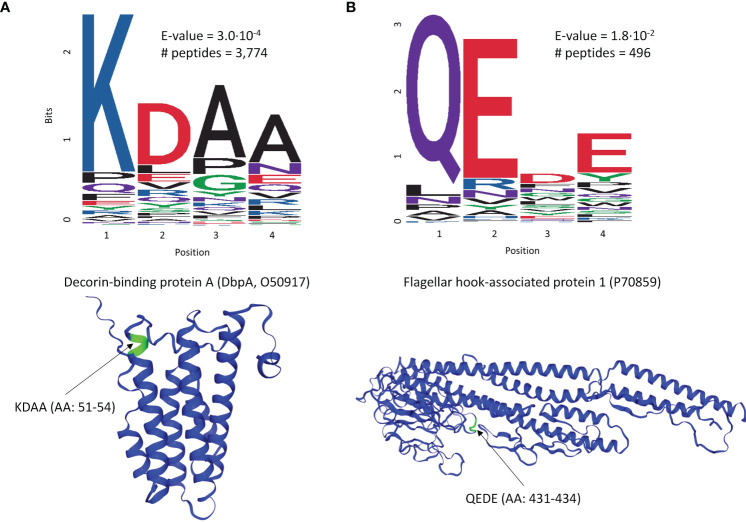
Motif discovery analysis results using the peptides selected by the classifier in **Figure 2A** as important for differentiation between the seropositive LD and non-endemic healthy controls. The motif length was limited to a maximum of 6 AAs. **(A)** The top motif ranked by the p-value and its location on decorin-binding protein A (DbpA), a well-characterized immunogen in LD **(B)** The second top ranked motif (QEDE) and its location on flagellar hook-associated protein 1 from the *B*. *burg.* proteome, the only protein containing the QEDE motif. E-values are p-values adjusted for multiple hypotheses comparison. # peptides denotes the number of peptides containing the motif.

To explore whether there are preferences among the Ab binding profiles for specific patterns (motifs) in the peptide sequences, a motif discovery analysis using the MEME STREME tool was performed (48). This analysis is based on the discovery of repeating patterns in the peptide sequences that the patient’s antibodies bind to. The algorithm finds patterns in a target set of sequences with statistically enriched representation compared to the control dataset (the full peptide library used in this study). In the context of a polyclonal Ab response that typically takes place following the infection with a pathogen, the presence of preferred binding to well defined motifs would suggest a more specific Ab targeting of antigens by the immune system. To perform motif discovery analysis, a total of 13,490 peptides selected by the XGBoost classifier as features with most differentiating power to distinguish seropositive LD from non-endemic healthy controls (**Figure 2A**) were used. No pre-selection of peptides was performed prior to training the classifier allowing the classifier to freely pick the peptides that best differentiate between the two cohorts. The MEME algorithm was run to discover contiguous motifs with a length of 3-6 amino acids using the set of the selected peptides as target and the entire peptide library as reference dataset. The algorithm identified 2 motifs with statistical significance (**Figure 3**) and varying representation of distinct amino acids. The most statistically significant motif, KDAA (**Figure 3A** **),** showed a low E-value (p-value adjusted for multiple comparisons) of 3.0·10^-4^ and a total number of peptides containing the motif of 3,774 suggesting a strong prevalence of Ab binding in the seropositive LD cohort compared with the healthy non-endemic controls. Mapping the motif onto the *B.burg.* proteome revealed that the motif is contained in 5 different proteins (**Supplementary Table S1**). One of them, decorin-binding protein A (DbpA, Uniprot ID: O50917), is a strong immunogen (49, 50) with diagnostic value in LD (51). The location of the motif in DbpA (**Figure 3A**) shows it is surface exposed and accessible for binding. The motif with the second-lowest p-value (lower statistical significance) is a tetramer QEDE with a markedly lower significance (p-value of 1.8E-2, adjusted for multiple comparisons) compared with the first motif. It mapped to a single protein-flagellar hook-associated protein 1 (Uniprot ID: P70859), **Supplementary Table S1**. Its surface-close location (**Figure 3B**) is amenable to binding and is in a loop region of the protein. The two discovered motifs differ markedly in the number of peptides they occur in and the p-values. The data implies that there are at least two distinct immunogenic regions the immune system is reacting to with differential reactivity between the seropositive LD patients and non-endemic healthy control donors. This also demonstrates the utility of the approach in gaining a detailed insight into potential antigenic regions of *B.burg.*

### Using peptide array binding and machine learning models to predict binding to *B. burgdorferi* proteome

While information about binding to the array peptides can be used for classification and binding motif discovery, it provides only limited biological insight into actual antigens targeted by the humoral immune response.

**Figure 4:**
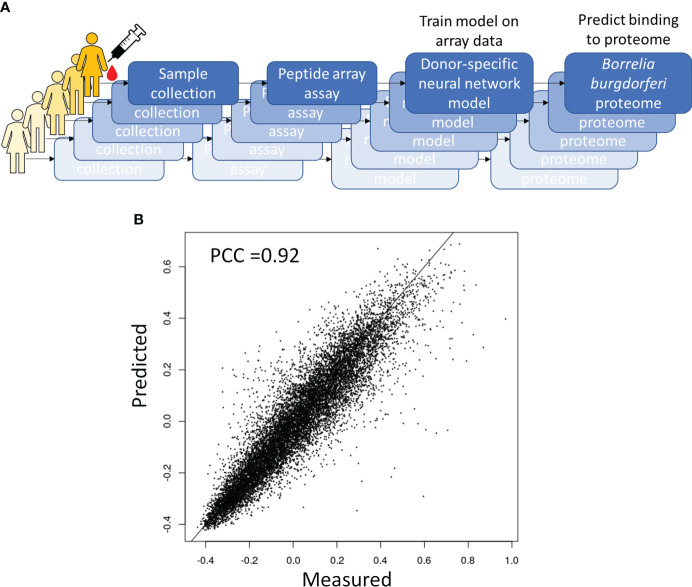
Predictive modeling of the immune response to *B*. *burg* infection. **(A)** Overall approach to predictive modeling of binding intensities for tiled proteins from B burg. proteome **(B)** Strong predictive performance of a deep learning regression model relating sequence to binding that was trained on anti-IgG binding to a diverse peptide array. Predicted binding intensities in a test dataset held out during training were compared to measured intensities. PCC-Pearson correlation coefficient.

One way to utilize the biological value of the sequence information is to instead use the binding information on the peptide array to train a model that mathematically relates sequence with binding intensity (**Figure 4A**). Such a model enables one to predict binding intensities to any peptide. It is then possible to apply this model to predict Ab binding intensities to tiled peptides from an entire proteome, e.g. the *B. burg*., and build classifiers based on these values. To achieve this, a separate neural network model was trained on the data from each donor to predict binding intensity of any peptide sequence of a set length of 10 AAs to ultimately predict antigens from the proteome with high reactivity to the patient’s antibodies. To this end, a feed-forward backpropagating NN model consisting of 3 hidden layers with 100 nodes each was used. Each hidden layer was implemented with batch normalization, a 10% dropout rate, and no bias. The models were trained with 25-50 epochs using a batch size of 256 data points. As a check of model performance, NN models were trained on randomly chosen 90% of all peptide intensity anti-IgG binding data and predicted the binding values of the remaining 10% left-out portion with 10 rounds of training and validation. For each donor one individual NN model was developed separately. The deep learning models showed robust performance in predicting actual peptide array data that was left out during model training with Pearson correlation coefficients between the measured and predicted binding intensities of 0.82-0.92 (**Figure 4B**) indicating good model prediction generalization. To generate predictions of binding to full proteins or the entire *B. burg.* proteome, the sequences of each protein were tiled into peptides of 10 AAs by using a sliding window with a 9 AA overlap between two adjacent tiles. The proteome tiles were then used as inputs for the NN models to compute predicted Ab binding intensities. The performance of the separate models was evaluated as the ability to predict differential binding to known, biologically relevant acute LD antigens when comparing the seropositive LD and endemic healthy controls. The two cohorts were chosen for comparison due to the opposing serological testing results (positive for LD and negative for controls). While classification performance of the two sets of samples was low compared with other sample types, differentiating confirmed LD and endemic controls is mostly relevant in the geographic areas with high disease prevalence. Predicted binding to a set of selected antigens that includes the 10 IgG antigens used in the STTT serological LD test was analyzed (**Table 2**). The STTT for LD recommended by the American Centers for Disease Control and Prevention (CDC) consists of 2 tests with test 1 (tier 1) based on enzymatic immunoassay (EIA) of Ab response to either a peptide from or the full VlsE protein (3). If the tier 1 test result is positive or indeterminate, a second test (tier 2) based on Western Blot (WB) assays using a panel of 3 biomarker proteins for the IgM Ab isotype and a panel of 10 biomarker proteins for the IgG Ab isotype (**Table 2**), is recommended. **Table 2** shows the false discovery rate (FDR) values of the predicted most significant tiles of the shown proteins for a total of 13 of known antigen LD proteins, including the 10 STTT biomarkers, predicted by the NN models when comparing the seropositive LD *vs* endemic healthy cohorts. The data show that all the STTT IgG proteins showed significantly higher predicted binding in the seropositive LD cohort compared with the samples in the endemic controls. Furthermore, we find that our predictions recapitulate the significantly stronger binding to the VlsE protein in the seropositive LD patients compared with the endemic controls.

**Table 2:** The model accurately predicts significantly different binding to all IgG antigens from the panel of 10 proteins used in the STTT clinical assay when comparing the confirmed LD cases *vs*. the endemic controls.

Protein	UID	STTT biomarker	FDR
DbpA	050917	×	6.21E-05
OspC	Q07337	×	2.09E-04
OspD	H7C7Q2	×	1.31E-04
p30	P70831	×	5.97E-07
OspA	POCL66		6.51E-04
OspB	P17739		3.03E-05
BmpA	Q45011	×	3.06E-04
Fla	P11089	×	4.09E-04
BBK32	050835	×	3.05E-04
HSP60	POC923	×	3.78E-05
P66	H7C7N8	×	4.06E-05
P83/100	Q45013	×	4.66E-05
VIsE	G5IXI6		8.07E-05

Using the same modeling approach and classification methods as explained above for the measured peptide array binding data (**Figure 2**), classifiers were trained on the values of predicted binding to the tiled *B.burg.* proteome. A comparison between the classifiers trained on the measured binding values on the peptide array and predicted binding to *B. burg.* proteome (**Table 3**) shows that in most cases the classifiers trained on the predicted data show similar to or better performance than the models trained on the peptide array binding data. Only the seropositive LD *vs* non-endemic healthy classification was slightly lower (0.90 *vs* 0.96) when using a model trained on the predicted binding values. This suggests that the predictive models used to project the peptide array data onto the *B.burg.* proteome maintain the cohort-specific information contained on the arrays. In addition, when comparing look-alike diseases with LD or endemic healthy controls, projection on the *B.burg.* proteome slightly increases classification performance suggesting that pathogen-specific information supplied in form of specific sequences can further improve differentiation. It is interesting to note that the low differentiating power of either seropositive or seronegative LD *vs* the endemic healthy controls persisted regardless of whether the peptide array itself or the predicted binding to the tiled *B.burg.* proteome was used (**Table 3**). Consistent with the clinical testing data, this result suggests a less pronounced humoral immune system response in the seronegative LD than in the seropositive LD cohort.

**Table 3:** Performance comparison between classifiers trained on measured peptide array data and predicted binding to tiled *B.burg.* proteome.

Contrast	Data used for training	Performance (AUC (95 CI))
Sero+ LD vs. NE healthy	Peptide array	0.96 (0.93-0.99)
	Bburg proteome	0.90 (0.86-0.94)
Sero- LD vs. NE healthy	Peptide array	0.92 (0.87-0.98)
	Bburg proteome	0.91 (0.85-0.97)
Combined Sero+/- LD vs. look-alike diseases	Peptide array	0.93 (0.91-0.95)
	Bburg proteome	0,97 (0.94-0.99)
Look-alike diseaeses vs. endemic healthy	Peptide array	0.96 (0.95-0.97)
	Bburg proteome	0.99 (0.98-1.00)
Sero+ LD VS. endemic healthy	Peptide array	0.82 (0.74-0.88)
	Bburg proteome	0.72 (0.65-0.79)
Sero- LD VS. endemic healthy	Peptide array	0.61 (0.54-0.68)
	Bburg proteome	0.57 (0.54-0.61)

NE, non-endemic.

### Evaluating epitope predictions based on 3D protein structure

**Figure 5:**
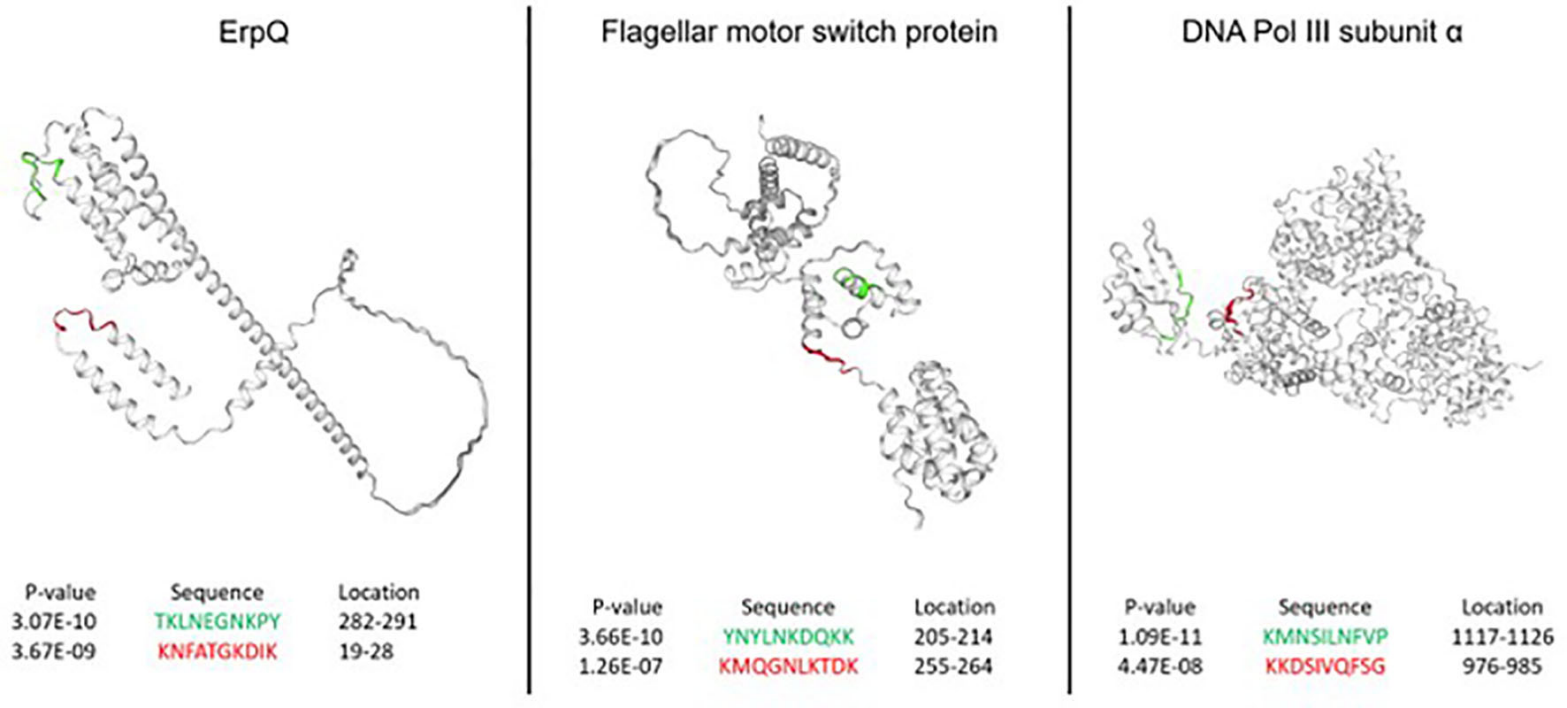
Epitopes in the B. burg. proteome with high predicted immunogenicity based on a NN model trained on the binding data from the diverse peptide array and comparison between seropositive LD and endemic healthy controls. The predicted antigenic regions are located on the outside of the proteins in the areas accessible to binding by antibodies. The predictions were produced using a tiled B. burg. proteome with a tile size of 10 amino acids. The p-values shown were calculated by performing the Welch’s t-test comparing the seropositive LD and endemic healthy controls.

Next, the performance of the NN models was assessed from the standpoint of predicting peptides in the accessible regions of the proteins from the *B. burg.* proteome. More specifically, it was explored whether the predicted epitopes identified as having the most differentiating power tend to be located in the protein regions generally accessible to Ab binding. While Ab binding may not be restricted to regions on the surface of the target proteins, for bacterial infections this mode of antigen recognition likely dominates. Three proteins were selected for this analysis: DNA polymerase II, ErpQ and Flagellar motor switch protein. DNA polymerase II subunit α (Uniprot ID: O51526) is the protein with the highest-ranking peptide by p-value (lowest p-value) when compared between the seropositive LD and endemic healthy controls. ErpQ (Uniprot ID: Q9S035) and Flagellar motor switch protein (Uniprot ID: O51239), were selected because they are known antigens that contain peptides with the highest ranking (lowest p-value). For each of the selected proteins, the highest-ranking peptide and the second-highest ranking peptide were mapped on the 3D structures of the proteins (**Figure 5**). A comparison of the spatial location of the peptides on the 3D structure of the 3 proteins showed that all 6 predicted antigenic peptides are located on the outer regions of the proteins and are at least partially accessible to Ab binding. All 6 predicted epitopes map primarily to the linear/loop-like regions of the proteins, with only the first epitope of flagellar motor switch protein showing partial overlap with a α-helix. This suggests that, at least in the 3 proteins analyzed here, there is a preference of the predicted epitopes to loop-like structures. As the models are trained purely on sequence-intensity relationships without any structural inference, the predictions are not based on structural aspects of the predicted epitopes. It is interesting to note that the relative locations of the predicted epitopes vary depending on the protein. While the epitopes are located relatively closely in terms of the physical location on the protein for flagellar motor switch and DNA Pol III subunit α, the epitopes of ErpQ are markedly farther (>250 AA) apart with one epitope being close to the N-terminus of the protein and the other close to the C-terminus. Despite the relative physical vicinity of the epitopes in the former two proteins, sequences of all epitopes have little similarity suggesting distinct immunogenic regions in the protein. Similarly, for ErpQ, the models predict epitopes with markedly different sequences. The lack of similarity among the epitope sequences suggests that the models predict at least two distinct antigenic regions in each of the proteins.

### Comparing predicted Ab reactivities to VlsE surface lipoprotein antigen between cohorts

**Figure 6:**
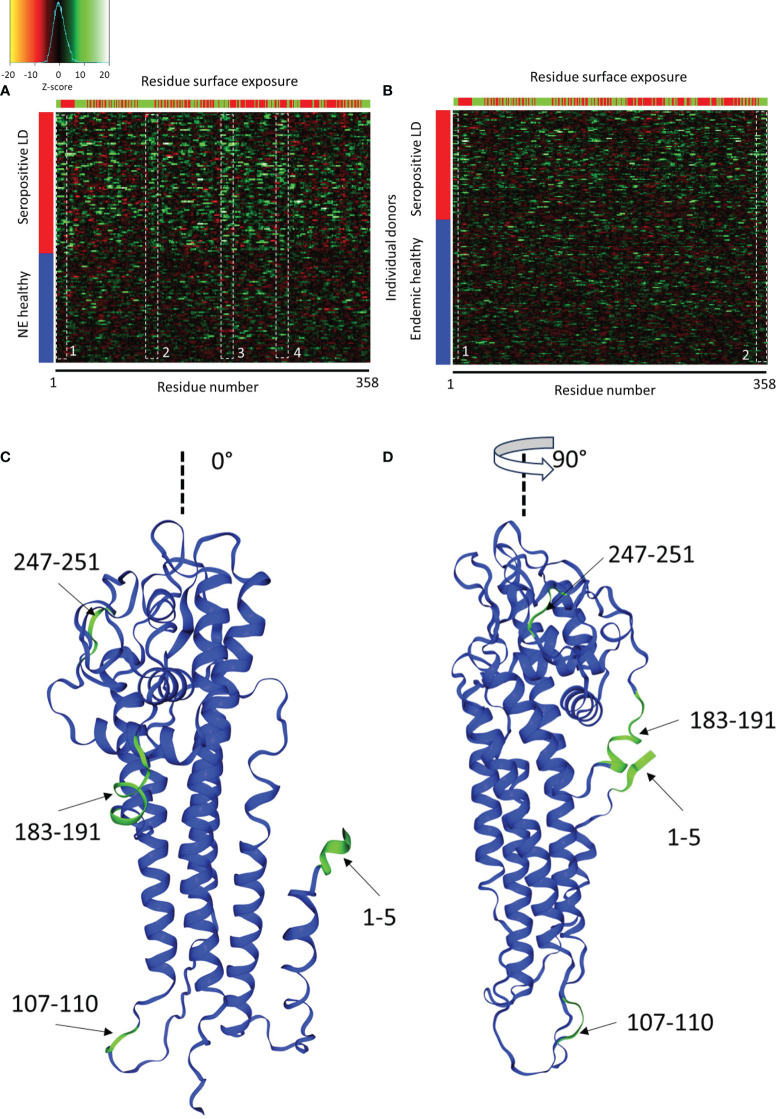
Comparison of Ab binding reactivity to the VlsE protein between the seropositive LD and non-endemic (NE) controls **(A)** and seropositive LD and endemic controls **(B)**. The data represented in the heatmaps are Z-scores computed with respect to the corresponding control cohort and calculated using a sliding sum window of 5 residues. Each row represents a single donor, while the columns are the amino acid residues from the protein sequence. Left color bar-cohort assignment, upper color bar – residue exposure with red representing hidden and green exposed to solvent residues. The dashed white boxes denote areas of partial overlap in binding reactivities among the different donors. **(C, D)** depict the relative positions of the four reactive regions in panel A on the 3D structure of the VlsE protein at 0° and 90° relative presentation angle, respectively (PDB: 1L8W, Uniprot ID: G5IXI6).

The VlsE protein is a known LD antigen and is used as a biomarker in the STTT. To gain insight into how variable the humoral immune response is in LD, relative reactivities to the tiled VlsE protein between the seropositive LD and endemic and non-endemic controls were compared. To this end, the protein amino acid sequence was broken down into tiles of 10 AAs with 9 AA overlap between two adjacent tiles. The relative reactivities for each donor in a cohort were computed as a Z-score using the endemic or non-endemic control cohort as a reference for calculating the mean and standard deviation values. First, the Z-scores were calculated for each tile of the protein (Equation 1).


(1) $$z_{i,j}=\frac{\left(x_{i,j}-\mu_{i,j}\right)}{d_{i,j}},$$

where i and j denote i^th^ tile of j^th^ protein, z is the z score, x_j_ is the predicted binding intensity, µ is the mean binding intensity, and d is the standard deviation of the same tile in either the endemic or non-endemic healthy controls. A comparison between the seropositive LD and the non-endemic healthy donors showed strong differentiation between the two cohorts (**Figure 6A**) with the seropositive LD samples exhibiting an overall stronger reactivity to the VlsE protein than the non-endemic controls. Interestingly, there are at least several distinct antigenic regions of the protein that overlap across the majority of the LD samples. However, the different regions appear to vary in terms of the samples where they are reactive. For example, region 1, demarkated by the white dashed box between residues 1-5 (**Figure 6A**) is strongly reactive in ~50% of the LD samples, whereas reactivity in regions 2 and 3 between residues 107-110 and 183-191, respectively, appears in samples that only partially overlap with those in region 1. Region 4 between residues 247-251, that overlaps partially with the invariable region 6 (IR6) of the VlsE, is reactive in ~40% of the LD samples. The comparison of the seropositive LD samples with the endemic healthy samples revealed a markedly different pattern (**Figure 6B**). with a substantially lower differential binding between the cohorts and only two somewhat discernable regions with increased Ab reactivity. In this comparison, the LD samples exhibit a more dispersed differential reactivity to the VlsE protein with little overlap between the samples. The region at the N-terminus (residues 1-10) shows increased reactivity in the LD cohort, although in only ~20-25% of samples. Similarly, a weakly reactive region can be seen at the C-terminus of the protein, also in about 20% of samples. The data shows that despite the presence of the overlapping regions of reactivity among the LD patients, there is marked overall variability in protein regions each donor responds to.

The predicted reactive regions were compared with the relative solvent accessibility (RSA) scores computed for each residue of the protein. A residue was considered “exposed” if its RSA score (fraction of the total residue volume accessible to solvent) was >25% and “hidden” otherwise. As can be seen in **Figure 6A** (seropositive LD *vs*. non-endemic healthy controls), reactivity regions 1-3 contain mainly exposed residues. In contrast, region 4 contains markedly more “hidden” and solvent-inaccessible residues. Region 4 also contains the invariable region 6 (IR6) of the protein which is one of the biomarker immunogens used currently in the serological testing for LD. IR6 has been demonstrated to be inaccessible to antibodies *in vivo*, which corresponds to the residue accessibility data. Mapping the common reactive regions onto the 3D structure of the VlsE protein (**Figures 6C, D**) confirmed the closeness of the regions to the surface and accessibility to binding. Three out of four reactive regions are close to the protein surface with reasonable accessibility to solution.

## Discussion

Lyme disease, a complex and difficult to diagnose condition caused in the US by the infection with the bacterium *B. burg.* was chosen as the subject of this study due to the known highly complex interaction between the bacterium and the humoral immune response. Combined with large person-to-person variability in immune system response, this complexity has so far prevented not only the development of reliable diagnostics for LD but also led to the gaps in knowledge about the interaction between the pathogen and dynamics in immune response.

This study was designed to apply a library of short linear peptides with a median length of 9 AAs and near-random sequences to sparsely, but uniformly sample the entire combinatorial sequence space of the same length peptides for profiling the humoral immune response to a *B.burg.* infection. Using machine learning and data from antibodies binding to library peptides, it is possible to reconstruct the binding/reactivity profile of the circulating Ab repertoire for an individual. Having characterized such a reactivity profile one can not only determine disease-specific antigens, but also gain broader understanding of disease pathology in terms of host immune response. The ability to reconstruct the binding profiles of a circulating Ab repertoire utilizing a sparse sample of the combinatorial binding space is based on the concept that there is a certain degree of promiscuity in Ab binding to their cognate epitopes. This promiscuity means that Abs raised against a specific immunogenic target can bind peptides with physicochemical properties resembling those of the cognate epitope, but with typically lower affinity. The binding strength should correlate with the degree of peptide similarity to the cognate epitope. As a result, one should be able to predict the cognate binding targets using information about Ab binding to such peptides. In this case, information about Ab binding to the peptides present on the peptide library representing only a relatively small sample of the binding space can be sufficient to learn about the underlying binding preferences via training of an appropriate model relating the sequence information in the library with the binding intensity values. This approach presumably works because the antibody binding information in this case is “distributed” among the entire library of the 10^5^ peptides, even though this represents a small sample of the entire combinatorial space of peptides. The sequence-binding relationship can then be used to predict binding to any sequence of the same or similar length as the training dataset, including entire tiled proteomes. As a result, it is possible to model the binding profiles of an Ab repertoire with statistical accuracy for a broad variety of applications ranging from biomarker discovery to potentially monitoring disease progression to response to treatment.

Because the peptide libraries used in the study represent a very sparse sample of the entire combinatorial peptide space, it is possible that binding of highly specific, high-affinity antibodies could be underrepresented on the array leading to potentially missing them altogether. However, our previous studies using monoclonal antibodies have indicated that even such highly specific interactions can still be resolved using the peptide arrays (44, 45), suggesting that the approach should be applicable to profiling of circulating antibodies that in general show less specific binding characteristics.

### Method comparison with previous studies

To validate the approach several analyses were performed to compare the findings of this work with the previous studies. The motif discovery revealed the presence of at least two short sequences with binding preference in the seropositive LD as compared to the non-endemic healthy controls (**Figure 3**). The most statistically significant motif KDAA was found to be part of decorin-binding protein A (DbpA), a strong immunogen in LD in animals (50) and humans (49) with diagnostic value (51). Interestingly, the motif was also found to be part of a highly immunoreactive peptide in LD patient sera in a study based on targeted peptide arrays (52). This finding provides strong support for the approach used in this study to discover biologically relevant immunogenic targets. While it is true that a motif of only 4 amino acids can be found in a number of proteins in e.g. human and the *Ixodes scapularis* tick proteomes, the finding that the two statistically significant motifs were identified using peptides with differentiating power between LD and non-endemic controls indicates pathogen, *B. burg.*, specificity.

The predictive ML models show strong predictive power for the test sets of sequences left-out from training on the bulk of the peptide array binding data (**Figure 4B**). The predicted Ab binding to the *B. burg*. proteome calculated using the ML models show good correspondence with previous studies in terms of statistically significant differential binding between the seropositive LD and endemic healthy control cohorts for the 10 biomarker antigens used in the STTT for serological testing (**Table 2**) (3). Taken together, these results demonstrate in broad terms the validity of the approach as an agnostic method for broad profiling of circulating Ab repertoire in patient’s sera.

### High donor-to-donor heterogeneity in antibody reactivity

The UMAP representation of the measured Ab binding to the library peptides intensities (**Figure 1B**) reveals a highly heterogeneous Ab response profile at the single-donor level. The data shows that all cohorts exhibit complex and partially overlapping distributions with no clear separation between them. The UMAP distribution of the data points indicates the presence of at least 3 clusters (dashed ellipses) that encompass all 5 cohorts to differing degrees. This suggests the presence of several sub-populations of the donors used in the study. The lack of cohort-specificity of the clusters suggests that, from the Ab reactivity perspective, donors from the same cohort, e.g. diagnosed with LD or healthy controls, are not confined within one single cluster. Ab binding in these donors can display patterns that are not only markedly different from other individuals in the same cohort but also partially overlap with donors from different cohorts. The degree of overlap differs among the donor types. The look-alike diseases separate most from the 4 other donor types with little overlap, whereas the LD and endemic healthy controls exhibit the strongest overlap. This suggests that the binding profiles show in broad terms high variability in Ab reactivity at the single-donor level within the same cohort. The high degree of heterogeneity in Ab binding between the individual donors is not unexpected. Because the peptide arrays represent a sparse and random probe of a much larger combinatorial binding space, the measured Ab binding patterns represent a broad screen of the entire Ab repertoire and not just the portion associated with an acute infection. In contrast to peptide arrays targeting a specific disease or pathogen, it is highly likely that the measured binding profiles encompass not only Abs raised in response to one specific pathogen (e.g. *B.burg.* in the confirmed LD donor cohort*).* Abs that were raised because of immune system responding to any additional pathogen(s) the body is also currently exposed to or has encountered in the past are also contributing to the binding pattern. Because the number of different pathogens a person has encountered during their lifetime can vary greatly between individuals, it is reasonable to expect that the broad profiling performed in this study would generally be highly variable at the single-individual level as well. To extract pathogen-specific information, the predictive ML models trained on the peptide array data were used to predict Ab binding to specific pathogen proteins. As an example, Ab binding characteristics to the VlsE protein from the *B.burg.* proteome, a known antigen with diagnostic value in LD, were analyzed. Here, binding predicted by the ML models revealed a highly variable binding profile at the individual donor level within the seropositive LD, endemic healthy and non-endemic healthy control groups. The seropositive LD samples that represent the strongest humoral immune response by the STTT standard to the pathogen among the cohorts, display predicted immunoreactive regions within the protein that are highly variable among the donors with only a partial overlap for several distinct regions of the protein (**Figure 6**). This finding is indicative of a heterogeneous humoral immune response to *B.burg.* among the infected individuals in terms of immunogenic targets, essentially resulting in little overlap in Ab reactivity among the patients. A possible explanation for this variability could be the molecular immunomodulatory and immunosuppressive tools deployed by the bacterium to prevent a strong humoral immune response from being adequately mounted by the host. High interpersonal variability in immune response in general (14, 15) and in response to *B.burg.* infection in humans in particular with B cell responses with little overlap between patients (53) have been reported previously and support the high person-to-person variability in humoral immune response observed in this study.

### Complexity in Ab binding profiles associated with LD

Comparison of Ab binding intensity profiles (**Figure 1A**) revealed 2 distinct peaks characteristic to all studied donor cohorts. The first peak is located at the low end of binding range and encompasses peptides that show little or no Ab binding. It is likely that the weak Ab binding to these peptides is a result of non-specific, low-affinity interactions between Abs and peptides. These interactions could be caused by hydrophobic properties or the presence of a charge in a peptide as a whole as opposed to its specific AA sequence. It is therefore not surprising to see the strong overlap of this part of the binding distributions across all cohorts. This result also indicates that, as one would expect with a random sampling of a large binding space represented by the patient’s circulating Abs, a substantial portion of the peptides does not show substantial binding. This is most likely due to the peptides being too dissimilar in their physicochemical properties to the cognate targets of the Abs. On the other hand, the second peak that represents the stronger binding peptides contains most of the Ab- and cohort-specific binding content. Both the position and the height of the second peak can vary substantially between the cohorts. In this regard, the look-alike diseases with etiology similar to LD and the non-endemic healthy controls display the largest differences whereas both LD cohorts (the seropositive and the seronegative/clinically diagnosed LD) and the endemic healthy controls are highly similar to one another as indicated by the strong overlap of the corresponding peaks. The varying position of the second peak with respect to the first across several cohorts is another interesting result. An increased shift of the second peak to the right in the plot (stronger binding) suggests an in general stronger and more distinct Ab reactivity and thus could be associated with a more pronounced humoral immune response. Accordingly, the proximity of the second peak to the first for the LD cases and the endemic healthy controls indicates a comparatively less-pronounced Ab response in LD cases than in look-alike diseases. A similar trend can be seen from the comparison of the distributions between the LD cases and the non-endemic healthy controls with the second peak shifted towards stronger binding in the latter.

Overall, a comparison of the cohort-level binding intensity distributions (**Figure 1A**) and the UMAP representations (**Figure 1B**) shows that circulating Ab repertoires of the seropositive, seronegative LD and the healthy controls from the LD endemic areas are similar to one another. In contrast, the non-endemic healthy and look-alike disease cohorts differentiate from the LD and endemic healthy controls. These results are further supported by the classifier models trained to differentiate between the conditions. The peptide array data allows for robust classification between the seropositive LD or seronegative LD and the healthy controls from non-endemic areas using classifiers with AUC values of 0.96 (95% CI: 0.93-0.99) and 0.92 (0.85-0.99), respectively (**Figures 2A, B**). The ability to reliably differentiate between the look-alike diseases and the combined (seropositive+seronegative) LD cohort (AUC 0.93 (95% CI: 0.91-0.95, **Figure 2C**) suggests that the Ab repertoires in the two types of samples are disease-specific. In addition, this shows that the linear peptide array binding data provides enough information to distinguish LD from other diseases with similar clinical symptoms. Furthermore, the finding that one can robustly differentiate between the look-alike diseases and healthy non-endemic controls (AUC 0.96 (95% CI: 0.95-0.97), **Figure 2D**) provides further support to the ability of the method to detect alterations in circulating Ab repertoire related to an ongoing immune response. In comparison, classification performance of the seropositive or seronegative LD *vs*. endemic healthy controls is markedly lower, resulting in AUCs of 0.82 and 0.61 (**Figures 2E, F**). This indicates that in the LD endemic geographic areas the immune response to *B.burg.* is less distinguishable from the healthy controls than the controls from non-endemic areas. The geography of the donors could be an important underlying factor. However, the finding that the non-endemic healthy controls and the look-alike diseases donors that cover a number of different geographic areas in the US, as opposed to the LD endemic area, are markedly different provides strong support that the differences are not geography-specific and are associated with the disease. The observed strong similarity between the seropositive and seronegative LD cohorts can be explained by the individual’s humoral immune response to the same pathogen (*B. burg.*). The lack of difference between the LD and the endemic healthy controls is somewhat unexpected and interesting. One possible explanation is that a significant portion of the currently healthy individuals living in the LD endemic areas had been exposed to the pathogen in the past and were either not diagnosed with Lyme disease or had asymptomatic disease. In such cases one could detect *B.burg*. specific Ab binding patterns potentially due to the presence of Ab-producing memory plasma B-cells in patient’s blood. The reported seroprevalence of Ab reactivity to *B. burg.* antigens in individuals exposed to tick bites can be as high as 38% (54) and could potentially act as a confounding factor when comparing the LD with the endemic healthy cohort. In addition, it is also possible that the immunomodulatory mechanisms used by *B.burg.* to weaken host immune response results in an overall suppressed Ab reactivity profile. The weak overall immune response to *B.burg.* observed in this study has also been reported in mice LD models (55) and human patients (53). A weakened humoral immune response could result in Ab binding in LD patients being similar to individuals from the endemic area that do not have an ongoing infection with the bacterium. These two factors, either in separation or combined, may contribute to the observed similar humoral response or lack thereof between the infected individuals and the endemic controls. The more distinct separation between the confirmed LD and non-endemic controls suggests that the Ab binding profile is different from the healthy individuals who are much less likely to have been exposed to *B. burg.* in the past. The strong differentiation performance between the LD and look-alike disease samples shows that the observed Ab response in the LD cohorts is different from other diseases, despite the apparent similarity between the LD and endemic controls.

Therefore, corroborating findings in the previous studies, this study demonstrates the complexity of LD with implications for the development of reliable serodiagnostic for the disease. The findings suggest that due to the potentially high prevalence of *B. burg*. specific antibodies in LD endemic areas, a set of unique biomarkers distinguishing active *vs*. previous infection is necessary to establish a robust diagnostic.

This study is limited in several different aspects. First, the use of only 16 out of 20 canonical AAs in the array peptides introduces a certain bias in binding data. Consequently, one is unable to sample the portion of the combinatorial space representing the 4 missing amino acids. While the effect of this incomplete sampling on the measured data is presently difficult to assess, it is likely to affect the NN model accuracy for binding predictions of epitopes in which the 4 missing AAs (methionine (M), threonine (T), isoleucine (I) and cysteine (C)) are critical residues. However, it should still be possible to derive useful information about the epitopes from mimotopes (peptides with sequences with physicochemical properties similar to the cognate sequence) that have one or more of the 4 missing AAs substituted with similar residues (e.g. M substituted with valine (V), T with serine (S) etc. based on the BLOSUM substitution scores). Second, due to the linear structure, the peptides on the array explore primarily binding to linear epitopes. This limits the applicability of the approach in discovering structural epitopes. As a result, the presented findings likely underestimate binding reactivities to immunogenic targets. Third, given the observed substantial heterogeneity in Ab reactivity, the sample sizes used in the study are comparatively small. This can limit the ability to identify antigens that appear only in a subgroup of individuals from a particular cohort. The number of samples was mainly dictated by sample availability. While the number of samples per look-alike disease category is low, the comparison was with the look-alike diseases pooled together as a single cohort, providing a larger effective size.

Despite these limitations, past studies conducted by this group suggest that the current approach can be used to differentiate humoral immune response to a number of pathogens (30–43). A more recent study using arrays of peptides with random sequences has demonstrated the feasibility of the method to reliably represent and distinguish a number of infectious diseases caused by viral pathogens (44) using sequence-binding information contained on the random display library. The findings of this study imply that the approach provides valuable information regarding not only the overall Ab binding profile on the peptide array for differentiating immune responses, but also with respect to the biologically relevant insights at the single-individual level in terms of antigens targeted by the humoral immune system in response to infection with the *B. burg*. bacterium.

In summary, the work has demonstrated that random planar peptide arrays combined with machine learning models not only provide a means for differentiating between different pathologies of a highly heterogenous and complex bacterial disease, but also enable the discovery of candidate biomarkers not previously known. Furthermore, the approach allows for deeper insight into overall humoral immune response in terms of circulating Ab repertoire at the single-patient level and is thus amenable to analyzing heterogenous immune responses. The fact that validated biomarkers provide statistically significant differential signals in the peptide array data indicates that it is possible to learn about binding preferences of Abs from relatively weak interactions with peptides randomly probing the combinatorial sequence space. This is in contrast with the biopanning approach where primarily strong interactions are being explored. This also supports the notion of polyclonal Ab response having enough promiscuity to allow for sufficient binding to peptides with similar physicochemical properties (mimotopes).

## Materials and methods

### Samples

Well-characterized LD patient and endemic healthy donor samples were obtained from the Lyme Disease Biobank (5). Samples were collected from patients with signs and symptoms of early Lyme and endemic controls. Samples were tested as described and categorized as seropositive Lyme having either an EM rash greater than 5 cm in diameter or PCR confirmation combined with positive STTT serology. The seronegative Lyme samples were obtained from patients having an EM rash greater than 5 cm in diameter, but without positive STTT serology. These patients were diagnosed with Lyme by a physician based on the clinical symptoms. The endemic healthy samples were collected from donors who reside in the LD endemic areas, are self-declared healthy and seronegative on the STTT. Participants were enrolled in East Hampton, NY, Central Wisconsin and Martha’s Vineyard, MA. Each of the three cohorts contained equivalent numbers of patients from each collection site. Cohorts and collection sites were balanced across each assay batch of microarrays. The patient samples for the diseases with similar etiology to LD as well as non-endemic healthy controls were obtained from several commercial sources (Boca Biolistics, Pompano Beach, FL, Discovery Life Sciences, Huntsville, AL, Creative Testing Solutions, Tempe, AZ, SeraCare, Milford, MA). Note that these samples were obtained from commercial biobanks with no data about their previous exposure to LD was provided. Given that the samples were collected outside of the LD endemic areas, it is likely that these patients have not been exposed to *B. burg.* infection.

### Peptide microarray assays

Peptide microarrays containing diverse peptides were synthesized in a commercial production facility (Cowper Sciences, Chandler, AZ), following a previously described library design and photo-lithography based manufacturing process (42, 46). Synthesis occurred on 200 mm silicon wafers which were diced into 25x75 mm microscope slide pieces each containing 24 arrays in a 3 x 8 pattern. Four slides could be loaded into a custom cassette enabling use of standard 96 well laboratory automation equipment. Microarrays used contained 125,509 diverse peptide sequences plus a set of 6,203 control peptides. The standard serum Ab profiling assay protocol described in Arvey et al. was used as modified for a modular research use assay system. All dilution and liquid handling steps were conducted on a BRAVO robotic pipetting station (Agilent, Santa Clara, CA). Samples were thawed from single use aliquots and diluted to 1:625 in assay buffer (PBST with 0.05% Tween 20, 0.1% Proclin 950 and 1% mannitol). Microarrays were rehydrated in distilled water for 1 hour (h), PBS for 30 minutes followed by assay buffer for 1 h. Diluted samples (90 ul) were applied to duplicate arrays and incubated for 1 h at 37°C with mixing (TeleShake95 platform mixer). The cassette was then washed three times in PBST-P using a 96 well microtiter plate washer (BioTek Instruments, Inc., Winooski, VT). Peptide bound serum antibodies were detected using either 4.0 nM goat anti-human IgG (H+L) conjugated to AlexaFluor 555 (Invitrogen-Thermo Fisher Scientific, Inc., Carlsbad, CA) or 4.0 nM Goat anti-human IgM (H+L) (Novus Biologicals, Centennial, CO), conjugated to DyLight 550 in secondary incubation buffer (0.5% casein in PBST-P) for 1 h with mixing at 37°C. After the final incubation, slides were washed three times with PBST-P followed by distilled water to remove residual salts. Slides were then sprayed with isopropanol and dried by centrifugation.

### Peptide microarray data extraction

Dried slides were imaged using an ImageXpress imaging system to detect fluorescently labeled secondary antibodies. The imager used an LED light engine (SemRock) centered at 532nm wavelength to excite fluorophore-conjugated secondary Ab. Mapix (version 7.2.1; Innopsys, Carbonne, France) was used to place a grid alignment file over the obtained images and extract the median foreground pixel intensities using the central 60% of each feature. Images were saved as TIFF files and extracted intensities saved as GenePix results files.

### Data quality checks

Images were inspected to identify arrays with artifacts and image anomalies. The samples associated with such arrays were re-assayed on arrays from the same production batch as the original assay. Since each slide contains 24 arrays, additional replicates of some samples were included on the re-assay batch to utilize the available arrays and ensure that each cohort was adequately represented on the individual slides.

### Modeling of peptide binding using machine learning

Predictive models were built using the machine learning methods based on feed-forward, backpropagating fully connected neural networks, similar to those described previously (44). Peptide sequence was one-hot encoded by transforming each peptide into a vector of length 256. The vector length was derived from a maximum peptide length of 16 residue positions with 16 possible amino acids for each position. The amino acid present at each position is represented as a 1 while all other elements of the vector, including empty positions, are zero. For encoding, all peptides are N-terminally justified as this is the free end of the peptide on the array (peptides are attached by the C-terminal). In prior tests, center or C-terminal justification yielded identical performance to N-terminal justification. Feed forward neural networks were constructed individually for each donor using R (version 4.2.2, R Foundation for Statistical Computing, Vienna, Austria) as programming language and utilizing TensorFlow (version 2.11.0) and Keras (version 2.11.1) as interface packages. The NN models were constructed using 3 hidden layers with 100 nodes each with a 10% dropout and no layer bias. RelU activation was used for each layer. Each NN model was trained 10 times using a random 90:10 split of the dataset each time. To compensate for occasionally imbalanced binding intensity distributions on the array showing lower numbers of strong binding peptides, the data points were weighted by the frequency of peptides appearing in an intensity interval. To this end, the entire intensity range was subdivided 100 equal bins and the number of peptides falling into each bin was calculated. The weight for each peptide was computed using the Equation 2:


(2) $$w_{i}=\frac{1}{\sqrt{n_{i}}}$$

where w_i_ is the weight of the i^th^ peptide and n_i_ is the number of peptides in the bin that the i^th^ peptide falls into.

Accuracy of the model to predict Ab binding to the array was evaluated by predicting the binding to the held out 10% of the data and reported as the Pearson correlation between the measured and predicted binding intensities. Binding to *B. burg.* epitopes was accomplished applying the NN models to the *B. burgdorferi* B31 reference proteome (Uniprot Accession # UP000001807) that had been represented as 10-mers with sliding window of one amino acid offsets.

## Data availability statement

The original contributions presented in the study are included in the article/**Supplementary Materials**, further inquiries can be directed to the corresponding author.

## Ethics statement

The requirement of ethical approval was waived by Arizona State University Institutional Review Board for the studies on humans because the sera samples used in the study were either completely de-identified or obtained from commercial repositories and are not considered human samples. The studies were conducted in accordance with the local legislation and institutional requirements. Written informed consent for participation was not required from the participants or the participants’ legal guardians/next of kin in accordance with the national legislation and institutional requirements. The human samples used in this study were acquired from a by- product of routine care or industry.

## Author contributions

LK: Conceptualization, Data curation, Formal analysis, Investigation, Methodology, Software, Writing – original draft. JL: Formal analysis, Investigation, Methodology, Writing – original draft, Writing – review & editing. NW: Conceptualization, Funding acquisition, Investigation, Methodology, Project administration, Supervision, Writing – review & editing.
